# Conversion to No-Till Improves Maize Nitrogen Use Efficiency in a Continuous Cover Cropping System

**DOI:** 10.1371/journal.pone.0164234

**Published:** 2016-10-06

**Authors:** Hazzar Habbib, Julien Verzeaux, Elodie Nivelle, David Roger, Jérôme Lacoux, Manuella Catterou, Bertrand Hirel, Frédéric Dubois, Thierry Tétu

**Affiliations:** 1 Ecologie et Dynamique des Systèmes Anthropisés (EDYSAN, FRE 3498 CNRS UPJV), Laboratoire d’Agroécologie, Ecophysiologie et Biologie intégrative, Université de Picardie Jules Verne, 33 rue St Leu, 80039 Amiens, Cedex, France; 2 Adaptation des Plantes à leur Environnement, Unité Mixte de Recherche 1318, Institut Jean-Pierre Bourgin, Institut National de la Recherche Agronomique, Centre de Versailles-Grignon, R.D. 10, F-78026 Versailles Cedex, France; USDA Agricultural Research Service, UNITED STATES

## Abstract

A two-year experiment was conducted in the field to measure the combined impact of tilling and N fertilization on various agronomic traits related to nitrogen (N) use efficiency and to grain yield in maize cultivated in the presence of a cover crop. Four years after conversion to no-till, a significant increase in N use efficiency N harvest index, N remobilization and N remobilization efficiency was observed both under no and high N fertilization conditions. Moreover, we observed that grain yield and grain N content were higher under no-till conditions only when N fertilizers were applied. Thus, agronomic practices based on continuous no-till appear to be a promising for increasing N use efficiency in maize.

## 1. Introduction

Maize (*Zea mays* L.), also known as corn, is an essential dual-use food and energy crop, both in terms of cultivated area and production of harvestable material. The yearly increase in maize productivity worldwide has been much higher compared to other cereals, being on average 60 kg per ha every year since 1960 [1]. The total world production of maize reached a record of 877×10^9^ kg in the 2011–2012 fiscal year [2]. Maize requires large amounts of nitrogen (N) inputs for optimum grain and silage production, due mainly to the ability of the crop to produce large quantities of dry matter [3–5]. However, in several studies it has been shown that increasing N fertilization above a certain threshold, neither leads to an increase in plant uptake nor in grain production [6–8]. N use efficiency (NUE), originally defined by Moll et al. [9] as the grain yield or biomass production yield obtained per unit of N available in the soil (already present and originating from fertilizer application), is inversely proportional to the amount of N fertilizer applied [10]. When the rate of N fertilization is too high, nitrate leaching occurs, leading to multiple damaging effects on the diversity and functioning of non-agricultural bacterial, animal and plant ecosystems [11,12]. In addition, fertilizer-derived N oxide emissions into the atmosphere contribute to the depletion of the ozone layer [13], whilst volatilized ammonia is returned as wet or dry deposition, which can cause acidification and eutrophication. Moreover it has been reported than an excessive application of N fertilizers can even lead to a decrease in grain yield [14,15]. Thus, improving NUE is particularly relevant for maize, for which global NUE has been estimated to be less than 50% [16,17].

Both from a physiological and agronomic point of view, NUE is the result of two main biological processes: N uptake efficiency (NUpE) which corresponds to the amount of N taken up per unit of available N, and N utilization efficiency (NUtE) which corresponds to the increase in biomass or yield per unit of N taken up [18,19]. During the plant developmental cycle, a number of complex physiological processes are involved in the control of plant NUE notably N uptake, N assimilation and N translocation [9,18–20]. Cereals in general and maize in particular, need to remobilize the N accumulated in proteins in vegetative tissues and at the same time take up and assimilate N after anthesis, in order to ensure storage protein deposition in the grain. In maize, both N uptake and N remobilization processes contribute equally to NUE [21], measurement of these two components was a major part of this study, which aimed at optimizing tillage practices for optimal soil N recovery.

It is well known that on top of mineral N fertilization, intensive mechanical cultivation practices such as tillage generally alters soil biological activity [22–24]. These intensive cultivation practices create compaction zones in the soil [25–27], expose the soil surface to wind and water erosion [28,29] and alter the soil organic matter (SOM) decomposition rate. Reicosky and Archer [30] reported that larger amounts of CO_2_ were released into the atmosphere as the result of tillage, which, in turn reduced the soil carbon (C) content. In contrast, conservation tillage practices under continuous cropping systems are known to improve SOM content [31–36], notably by enhancing C accumulation in soil aggregates. Hence, compared to conventional tillage, agricultural practices based on the use of conservation tillage are in many cases beneficial in terms of crop yield improvement [37–39].

Moreover, it has been shown that both under tilling and no-till cultivation conditions, the use of cover crops captures the excess mineral N remaining in the soil during winter and early spring periods, thus limiting the amount of mineral N that can leach into ground water [40–42]. Furthermore, several studies have demonstrated that due to their ability to fix atmospheric N, legume cover crops have a beneficial impact on crop production [43–46] by increasing soil fertility, notably by increasing the N content [47].

A large number of studies have focused on improving N fertilizer management practices in order to increase both NUE and yield in many crops, including maize [48–51]. Among these management practices no-till has been increasingly used. However, its impact remains to be thoroughly characterized both in terms of plant NUE and plant productivity.

In the present study, maize plants were grown over four years in the presence of cover crops under tillage and no-till conditions to evaluate the combined effect of tilling and N fertilization on NUE and NUE-related traits. In the absence of mineral N fertilization, an increase in NUE and NUE-related traits including nitrogen remobilization (NRem), nitrogen remobilization efficiency (NRE) and nitrogen harvest index (NHI) under no-till conditions was observed. Thus, no-till appears to be a promising strategy for maintaining maize productivity without additional N fertilizers inputs.

## 2. Materials and Methods

### 2.1. Site Description and Experimental Design

The field experiment was conducted at the experimental site of La Woestyne, in North France (50°44′N, 2°22′E, 40 m a.s.l.). The owner of the land "Bonduelle company" gave permission to conduct the study on this site. The field studies did not involve endangered or protected species. Physical and chemical soil characteristics are presented in Table 1. Weather-related parameters for this area are as follows: average annual rainfall 675 mm, average annual temperature 10.5°C.

**Table 1:** Characteristics of the soil used for evaluating the impact of no-till and N fertilization on maize NUE at the beginning of the experiment in 2010.

Parameters (units)	Depth range (cm)	Value
Clay <2 μm (g kg^-1^)	0–30	211.6
Silt 2–20 μm (g kg^-1^)	0–30	232.3
Silt 20–50 μm (g kg^-1^)	0–30	436.3
Fine sand 50–200 μm (g kg^-1^)	0–30	95.2
Coarse sand (200–2000 μm) (g kg^-1^)	0–30	24.6
pH in H_2_O	0–15	6.9
CEC^a^ (cmol^+^ kg^-1^)	0–15	12
P^b^ (mg kg^-1^)	0–15	24
Organic Carbon (g kg^-1^)	0–15	11.6
Exchangeable cations (cmol^+^ kg^-1^)		
Ca^2+^	0–15	17.5
Mg^2+^	0–15	0.83
Na^+^	0–15	<0.43
K^+^	0–15	0.77
Penetration Resistance (MPa)		
With a soil moisture of 33%	0–15	0.7
	15–30	1.1
	30–45	1.7
	45–60	2.2

^a^ Cation-exchange capacity (Metson method)

^b^ Available phosphorus (Olsen method)

The field was managed under a chisel plough and rotary power system until 2010, when the experiment was initiated. The field experiment was split into four treatments with three replicated plots placed randomly for each of the four treatments including: no-till with (NTN1) or without (NTN0) N fertilization; conventional tillage with (CTN1) or without (CTN0) N fertilization. The individual plot size was 7m×8m for each treatment. Since the beginning of the experiment in 2010, the conventional tillage in CT plots was performed using the moldboard plowing technique followed by the passing of a rotating harrow (Kuhn, France) for shallow tillage (30 cm tillage depth). In 2013 (3 years after the beginning of the field experiment) and in 2014 (4 years after the beginning of the field experiment), maize samples were collected in each plot in 2013 and in 2014. The crop rotation preceding maize cultivation in 2013 consisted of green bean (*Phaseolus vulgaris* L.) in 2010, followed by wheat (*Triticum aestivum*) in 2011, pea (*Pisum sativum*) in 2012. In the maize culture performed in 2014, the crop rotation consisted of wheat (*Triticum aestivum*) in 2010, followed by green bean (*Phaseolus vulgaris* L.) in 2011, wheat (*Triticum aestivum*) in 2012, pea (*Pisum sativum*) in 2013 (S1 Fig).

Before sowing the main crop, cover crop residues were buried in CT plots and left on the soil surface in NT plots. This cover crop consisted of a mixture of legume and non-legume species which were sown as follow: 12 kg ha^-1^ of Egyptian clover (*Trifolium alexandrinum* L.), 100 kg ha^-1^ of faba bean (*Vicia faba* L.), 20 kg ha^-1^ of vetch (*Vicia sativa* L.), 5 kg ha^-1^of flax (*Linum usitatissimum* L.), 4 kg ha^-1^ of phacelia (*Phacelia tanacetifolia* Benth.), 10 kg ha^-1^ of oats (*Avena sativa* L.). To evaluate N inputs from cover crops residues, in each of the four plots, 3 × 1 m^2^ of cover crops were sampled each year. Samples were dried in an oven at 65°C for three days and then weighed. The total aboveground biomass of cover crops was ground into powder prior total N measurements. From the beginning of the experiment in 2010, means of N input originating from the cover crops residues in each of the four treatments were (123 kg ha^-1^, 127 kg ha^-1^, 125 kg ha^-1^ and 128 kg ha^-1^) under NTN0, NTN1, CTN0 and CTN1 conditions respectively.

The amount of N fertilizer applied under N1 conditions was determined according to the N budget method for maize [52], based on the predictive balance-sheet method (Software Azobil, INRA, Laon, France) using the following formula:

B+Rf=(Ri-L)+Mn+X

Where B is the N requirement of the crop, Rf is the residual soil mineral nitrogen content at harvest, Ri is the readily available soil mineral nitrogen in a determined depth of soil before maize planting, L is the soil mineral nitrogen potential loss during the period from analysis of soil N to N-fertilizer application, X is N the fertilizer rate and Mn is the net supply of soil mineral nitrogen during the growing season. Mn results from the sum of the net mineralization from SOM, the mineral N supply from previous crop residues and the mineral N supply from organic manures. All the terms are expressed in kg N ha^-1^.

The final amounts of N fertilizer applied under N1 conditions were 97 kg N ha^-1^ in 2013 and 80 kg N ha^-1^ in 2014. The N fertilizer was composed of 50% urea, 25% ammonium, 25% nitrate applied in a liquid form on the soil surface through broadcast applications at daybreak or at nightfall. Under these conditions of application, it was assumed that N volatilization was negligible.

### 2.2. Soil Sampling and Chemical Analyses

In March 2013 and 2014, six 30-cm deep soil cores were randomly collected using a 2-cm diameter auger in each of the three replicated plots for the four treatments (NTN1, NTN0, CTN1 and CTN0). Six soil cores from each replicate plot were collected and pooled, thus forming a single sample in each of the three replicates. Soils were then sieved using a 2 mm mesh and divided into two parts, for soil total N and soil residual N analysis. For soil total N measurements, the sieved soil was dried in an oven at 45°C for 48 h and ball milled ground (MM 400, Retsch, Germany). Soil total N (expressed as % of dry soil) was quantified using the combustion method of Dumas [53] using a Flash EA 1112 elemental analyzer, Thermo Electron, Germany.

Residual N (expressed in kg N ha^-1^) corresponds to the N originating from nitrate and ammonium present in the soil. Nitrate and ammonium were extracted using 20 g of fresh soil mixed with 100 mL of 1 M KCl. After shaking for 1 h, the soil extracts were centrifuged for 10 min at 4,000 *g* and the supernatant was analyzed using a continuous flow analytical system (San^++^ system, Skalar, Holland). The measured amounts of total N and residual N present in the soil before maize sowing in April 2013 and 2014 are shown in Table 2.

**Table 2:** Soil total N (%) and Soil residual N (kg ha^-1^) under two tillage systems and N fertilizer rates in the two studied years.

Year		2013		2014	
N fertilizer	Tillage	Soil total N (%)	Soil residual N (kg ha^-1^)	Soil total N (%)	Soil residual N (kg ha^-1^)
N0	NT	0.26	50.07	0.26	44.66
	CT	0.25	64.40	0.24	64.29
N1	NT	0.27	79.55	0.26	52.98
	CT	0.28	63.80	0.27	48.21

NT = No-till with cover crops, CT = Conventional tillage with cover crops, N0 = no fertilization, N1 = N fertilization

Soil water content (%) at sowing in April (SWC.s) and at crop harvest (SWC.h) in October were determined by using a moisture meter connected to a Penetrologger (Eijkelkamp, The Netherlands).

### 2.3. Crop Sampling and Plant Analysis

Maize (*Zea mays*, var. SY Cookie, Syngenta, Switzerland) was sown in 75 cm spaced rows using a Kuhn Maxima drill (Kuhn, France). At anthesis and at crop maturity when both stover and grains were dried [54], 6 rows of 1m length were sampled in 2013 and 2014 in each of the four treatments (NTN1, NTN0, CTN1 and CTN0). The shoots were clipped at ground level and threshed to separate the grain for yield per m^2^ measurements. Shoots and grain were dried in an oven at 60°C for 3 days, weighed and finally ground in a Retsch mill (Retsch zm200, Haan, Germany) to obtain a fine powder (0.75 mm particles). Grain and stover N contents were quantified using the same elemental analyzer as that used for soil N content analysis.

Traits related to NUE were calculated according to Moll et al. [9], Huggins and Pan [55] and López-Bellido et al.[49] using the following equations:-

$$\text{NUE}\;\left({\text{kg kg}}^{\text{-}1}\right)=\text{Gy}/\text{Nsupply}$$ (1)

$$\text{NUtE}\;\left({\text{kg kg}}^{\text{-}1}\right)=\text{Gy}/\text{Nt}$$ (2)

NHI(%)=(Ng/Nt)×100 (3)

where, Gy corresponds to grain yield (kg ha^-1^), Nt to total plant N at maturity (kg ha^-1^), Ng is the grain N (kg ha^-1^) and N supply, the soil N available to the crop (expressed in kg kg^-1^).The available N corresponds to the sum of applied N fertilizer and of total plant N uptake in non-fertilized plots in the tilled and no-tilled cultivation systems [6]. To measure the amount of N remobilized from vegetative to reproductive organs after anthesis (NRem), the following equations were used according to the method described by Cox et al. [56], Beheshti and Behboodi [57] and Masoni et al. [58]:

$$\text{NRem}\;\left({\text{g plant}}^{\text{-}1}\right)=\text{N content of the whole plant at anthesis - N content of leaves, stem and chaff at maturity}.$$ (4)

NRE(%)=(NRem/N content of the whole plant at anthesis)×100. (5)

### 2.4. Statistical Analyses

All statistical analyses were performed in R Statistical Software version 3.2.3 [59]. Data were subjected to variance analysis (Two-way ANOVA), using tillage practices (CT, NT) as the main parameters and the level of N application (N0, N1) as the second parameters. All explanatory variables were examined for normality using the Shapiro-Wilk test [60] and for homogeneity of variances with the Bartlett test [61]. Means of each of the four treatments (NTN1, NTN0, CTN1 and CTN0) were compared using Duncan's new multiple range test at a 95% family-wise confidence level (Agricolae package) [62]. Correlations between agronomical variables (grain yield, plant N, soil N, soil water content) and NUE-related traits (NUE, NUtE, NRem, NRE and NHI) were computed using a Pearson product-moment correlation coefficient at *P*<0.05 (Hmisc package) [63]. Principal component analysis (PCA) (ade4 package) [64] was also carried out to visualize relationships existing between NUE-related traits (NUE, NUtE, NRem, NRE and NHI) and agronomic traits (Grain yield, soil N total, plant N, SWC.s and SWC.h).

## 3. Results

### 3.1. Effect of Tillage on Agronomic and NUE-Related Traits

Grain yield over the two years of experimentation ranged from 8060.00 to 12757.33 kg ha^-1^ (Table 3). In 2013 and in 2014, grain yield was not significantly different between tillage and no-till conditions, whereas N fertilization significantly increased grain production both under NT and CT conditions (*P* < 0.001, *P* < 0.05 in 2013 and 2014 respectively).

**Table 3:** Impact of tilling and nitrogen fertilization on maize agronomic traits (mean ± standard error).

Source of variance			Agronomic trait			
Year	N fertilizer	Tillage	Total biomass (kg ha^-1^)	Total plant N uptake (kg ha^-1^)	Grain yield (kg ha^-1^)	N grain (kg ha^-1^)
2013	N0	NT	17817 ± 1534 *b*	166.53 ± 22.94 *b*	8060.0 ± 1064.7 *b*	108.02 ± 17.98 *b*
		CT	19833 ± 748 *b*	212.49 ± 6.08 *b*	9458.0 ± 517.4 *b*	118.44 ± 6.40 *b*
	N1	NT	24513 ± 1059 *a*	298.76 ± 29.86 *a*	12757 ± 895 *a*	186.06 ± 19.16 *a*
		CT	23919 ± 480 *a*	301.26 ± 11.74 *a*	11846 ± 315 *a*	159.59 ± 3.10 *a*
	2014	N0	NT	24578 ± 1891	252.22 ± 37.90 *c*	9676.4 ± 972.0 *b*	107.51 ± 14.68
		CT	30822 ± 1791	305.53 ± 22.34 *bc*	10408 ± 282 *b*	112.32 ± 5.43
N1		NT	29400 ± 2002	366.39 ± 37.74 *ab*	10074 ± 592 *a*	122.03 ± 7.71
		CT	32200 ± 1225	431.49 ± 17.67 *a*	10829 ± 582 *a*	130.82 ± 11.00
Analyse of variance			*P>F* (n = 6)			
2013	Tillage		ns	ns	ns	ns
	N fertilizer		<0.001 ***	<0.001 ***	<0.001***	<0.001***
	Tillage×N fertilizer		ns	ns	ns	ns
2014	Tillage		ns	ns	ns	ns
	N fertilizer		ns	<0.001 ***	<0.05*	ns
	Tillage×N fertilizer		ns	ns	ns	ns

NT = No-till with cover crops, CT = Conventional tillage with cover crops, N0 = no fertilization, N1 = N fertilization. Data for each parameter were subjected to variance analysis (Two-way ANOVA). Treatment means were compared using Duncan's new multiple range test at a 95% family-wise confidence level. Means with the same letter are not significantly different. (*, *** = significant at 0.05 and 0.001 probability level, respectively). ns = not significant.

Total biomass production was not significantly modified under CT or NT conditions over the two years of experimentation. In 2013, N application increased the total biomass production significantly, irrespective of the tilling conditions (Table 3).

Both in 2013 and 2014, total N uptake was not significantly modified under CT and NT conditions. However, N uptake was higher when N fertilizers were applied (N1 treatment) both in the tilling and no-till system (Table 3).

Tillage did not modify grain N content both in 2013 and in 2014. In contrast, when N fertilizers were applied, a significant increase (*P* < 0.001) in the grain N content was observed, only in 2013 (Table 3).

The ANOVA statistical analysis indicated that SWC.s and SWC.h were not significantly different between N0 and N1 (Table 4). In contrast, tillage had a significant effect on SWC both at sowing and at harvest both in 2013 and in 2014. A significant increase in SWC.s and SWC.h was also observed in NT compared to CT, only under N1 conditions.

**Table 4:** Impact of tilling and nitrogen fertilization on soil water content (%) at sowing and at crop harvest (mean ± standard error).

Source of variance		2013		2014	
N fertilizer	Tillage	SWC.s (%)	SWC.h (%)	SWC.s (%)	SWC.h (%)
N0	NT	38.83 ± 0.47 *a*	40.83 ± 0.94 *a*	39.33 ± 0.33 *a*	41.50 ± 0.76 *a*
	CT	37.00 ± 0.57 *b*	37.66 ± 0.49 *b*	34.50 ± 0.99 *c*	37.31 ± 0.66 *b*
N1	NT	38.16 ± 0.47 *ab*	39.66 ± 0.66 *ab*	38.16 ± 0.47 *ab*	39.33 ± 0.80 *b*
	CT	36.66 ± 0.21 *b*	38.00 ± 0.36 *b*	36.66 ± 0.21 *b*	38.00 ± 0.36 *b*
Analyse of variance	*P>F* (n = 6)				
Tillage		<0.01**	<0.01**	<0.001***	<0.001***
N fertilizer		ns	ns	ns	ns
Tillage×N fertilizer		ns	ns	<0.01**	<0.05*

NT = No-till with cover crops, CT = Conventional tillage with cover crops, N0 = no fertilization, N1 = N fertilization, SWC.s = soil water content at sowing, SWC.h = soil water content at harvest. Data for each parameter were subjected to variance analysis (Two-way ANOVA). Treatment means were compared using Duncan's new multiple range test at a 95% family-wise confidence level. Means with the same letter are not significantly different. (*, **, *** = significant at 0.05, 0.01, 0.001 probability level, respectively). ns = not significant.

**Fig 1:**
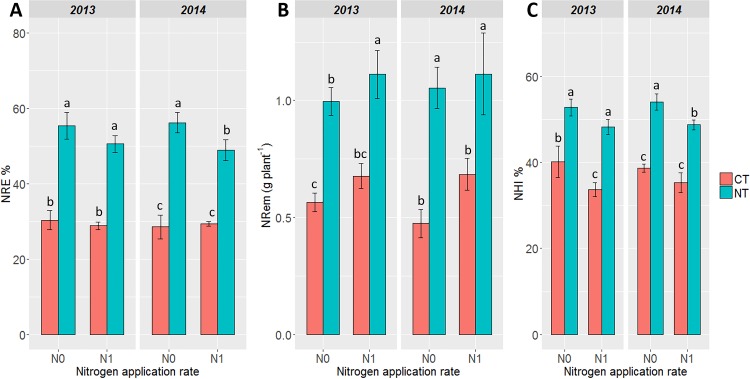
**Impact of tillage practice and N application on (A) NRE; (B) NRem and (C) NHI, according to the soil tillage treatment in 2013 and 2014**. (NT) No-till with cover crops, (CT) Conventional tillage with cover crops. N0 = no fertilization, N1 = N fertilization. Data for each parameter were subjected to variance analysis (two-way ANOVA). Treatment means were compared using Duncan's new multiple range test at a 95% family-wise confidence level. Means with the same letter are not significantly different.

In 2013 and in 2014, tillage had a significant and negative impact on NRE compared to the NT conditions (*P* < 0.001 in both years) (Fig 1A). The application of N fertilizer increased NRE under NT conditions. However, N application did not increase NRE under CT conditions.

The tillage system had a significant effect on NRem. This positive effect was significantly higher (*P* < 0.001) under NT compared to CT (Fig 1B) in both years, whereas Nrem was not modified whatever the N the fertilization conditions.

In 2013, the N fertilization did not significantly modify NHI, either under NT or CT conditions. However, both in N0 or N1, tillage had a negative effect on NHI compared to the NT cultivation system over the two years of experimentation (*P* < 0.001, *P* < 0.05 in 2013 and 2014 respectively) (Fig 1C). The N fertilization significantly modified NHI under CT conditions in 2013 and under NT conditions in 2014.

**Fig 2:**
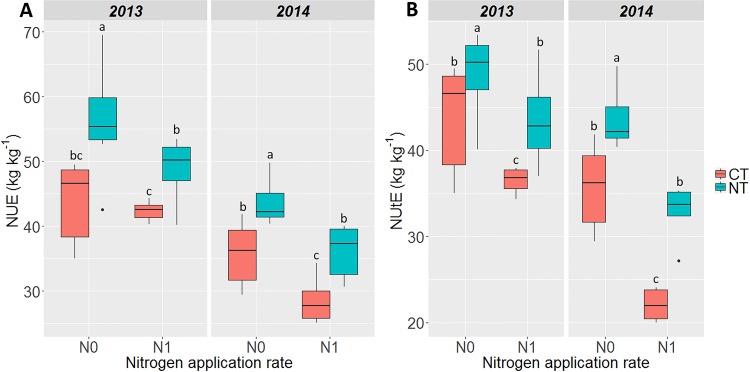
**Impact of tillage practice and N application on (A) NUE of maize grain (kg kg**^**-1**^**) and (B) NUtE of maize grain (kg kg**^**-1**^**) according to the soil tillage treatment in 2013 and 2014**. (NT) No-till with cover crops, (CT) Conventional tillage with cover crops. N0 = no fertilization, N1 = N fertilization. Box plots are represented with the median and the 25th-75th percentile with the minimum and the maximum. Data for each parameter were subjected to analysis of variance (two-way ANOVA). Treatment means were compared using Duncan's new multiple range test at a 95% family-wise confidence level. Means with the same letter are not significantly different.

Both in 2013 and 2014, CT and N application had a significant negative impact on NUE and its component NUtE (*P* < 0.001). Under N0 and N1, both NUE and NUtE were significantly higher in NT compared to CT conditions (Fig 2). A significant decrease in NUE and NUtE was also observed when N fertilizers were applied both under NT and CT conditions.

### 3.2. Correlation Analyses

**Fig 3:**
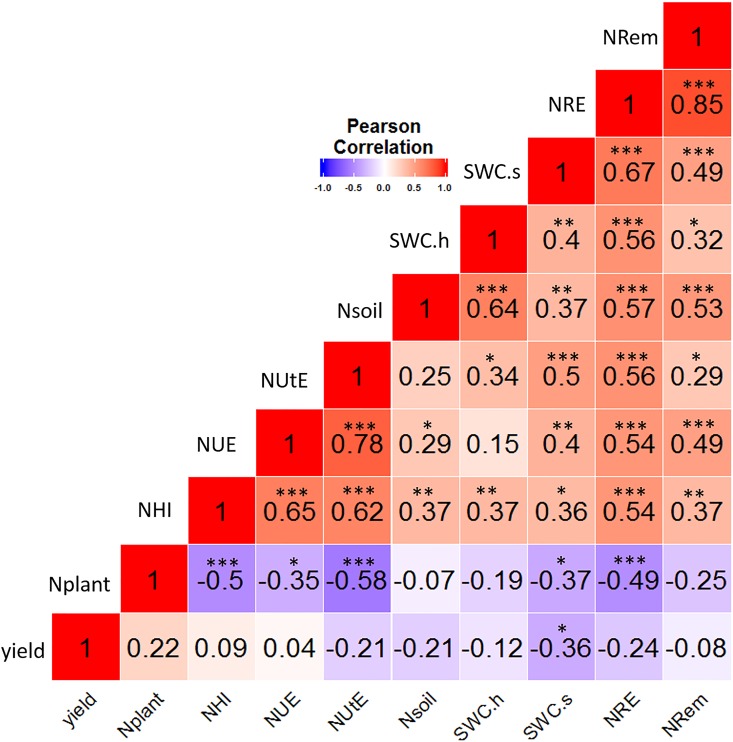
Pearson correlation coefficient *r* between NUE and NUE-related traits. (*, **, *** = significant at 0.05, 0.01, 0.001 probability level, respectively.).

**Fig 4:**
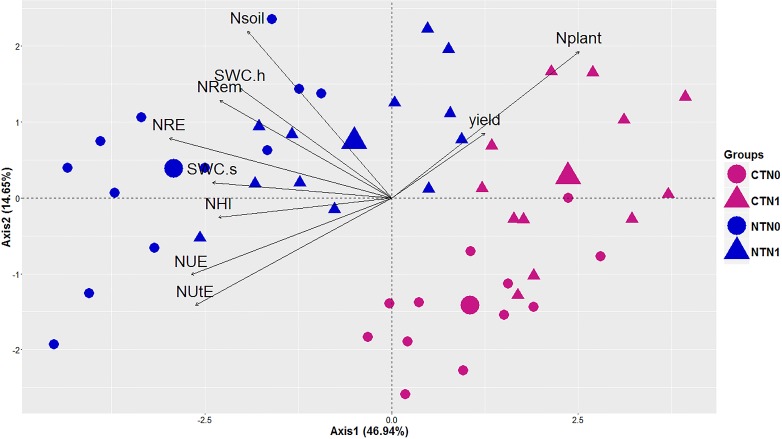
PCA analysis showing the correlations between tilling conditions, N fertilization and NUE-related traits. Diagrams were defined by the first two axes of the PCA of the variables (n = 12). Axis.1 (46.94% of variance explained) and Axis.2 (14.65% of variance explained). NTN0 = No-till without N fertilization, CTN0 = Conventional tillage without N fertilization, NTN1 = No-till with N fertilization, CTN1 = Conventional tillage with N fertilization.

Pearson correlations between NUE, NUtE, yield, soil N, total plant N, NRem, NRE, SWC.s, SWC.h and NHI over the two years of experimentation are presented in Fig 3. NUE and NUtE were significantly and positively correlated with NRem, NRE, NHI, soil N, SWC.s and SWC.h. Similarly, NRE, NRem and NHI were significantly correlated with the soil N content, SWC.s and SWC.h. Conversely, NUE, NUtE, NRE and NHI were significantly and negatively correlated with the plant N content. A PCA analysis was then performed to obtain a visual representation of the correlations between agronomic and NUE-related traits, according to the tillage system and the level of N fertilization (Fig 4). The first two axes of a PCA using NUE traits explained 61.59% of the variation in the data set. The variables were separated into four groups corresponding to tillage system and fertilizer application rate. Axis.1 (46.94% of variance explained) was positively correlated with plant N and yield, and negatively correlated with soil N, SWC.s, SWC.h, NRem, NRE, NHI, NUE and NUtE, which matches the Pearson correlation test. NRem, NRE, soil N, SWC.s and SWC.h were strongly correlated and positively grouped along Axis.2 (14.65% of variance explained). Similarly, NUE, NUtE and NHI were strongly correlated and negatively grouped along Axis.2. The first axis clearly separated the CT treatment from the NT treatment. The N0 and N1 fertilization conditions were separated along the second axis. NUE and NUtE related traits were markedly higher under NT conditions compared to the CT treatment.

## 4. Discussion

The field experiment performed over two consecutive years showed that conversion to no-till under a continuous cover cropping system significantly increased maize NUE and NUtE, in comparison to a cultivation system based on continuous till. Moreover, such an increase occurred both under low and high N fertilization conditions (Fig 2). These results are in agreement with those obtained with wheat by Soon et al. [65], who showed that NUE was increased under no-till conditions. In contrast, Brennan et al. [6] and López-Bellido and López-Bellido [7] found that in wheat, NUE was lower under reduced or no-till conditions respectively, likely because under their experimental conditions, crop N uptake was reduced. Another survey conducted by Dalal et al. [34] over 40 years of experimentation, led to the conclusion that wheat NUE remained constant, irrespective of the tilling practices employed. These contrasting results can be explained by the fact that in conservation systems, there is often an inefficient mobilization of N generated by plant residues left at the soil surface, thus leading to a decrease in NUE [66,67]. The originality of our study was to show that in maize, a crop rarely tested for its ability to valorize N under continuous till conditions, NUE is higher when the soil is not plowed, irrespective of the N fertilization regime.

In agreement with Burgess et al. [66] and Torbert et al. [68], grain yield, remained similar either under low or high N fertilization, regardless of the tilling conditions. In other studies, it has been reported that maize yields decrease slightly when no-till is used instead of conventional tilling, likely because the soil N availability is lower leading to a reduction in crop productivity [69,70]. In spring cereals such as barley, oats and wheat, it was generally observed that under no-till conditions, grain yield was substantially reduced [71–73]. Under the experimental conditions employed in these studies, the combined effect of tillage and of the level of N fertilization did not markedly modify N uptake, as slightly more N was taken up by the maize plants under CT conditions. Moreover, in agreement with Al-Kaisi and Kwaw-Mensah [74], we observed that such a small increase in N uptake did not lead to an increase in the grain N content (Table 3).

NUE is a complex agronomic traits depending on soil N availability, resulting from the efficiency of N uptake by the roots and N utilization and N remobilization by the plant [75]. Among the various traits representative of NUE, NHI was higher under NT conditions compared to CT, irrespective of the level of N fertilization. Similarly, other NUE-related traits such as NRem and NRE were significantly higher under NT compared to CT, whether or not N fertilizers were applied (Fig 1A and 1B). In addition, NHI and NRE were positively and significantly correlated with NUE and NUtE (Fig 3). Although the leaf N content at maturity was higher in CT than in NT, the grain N content was similar irrespective of the tillage practice (Table 3).

In this study, tillage had a negative impact on the amount of water stored in the soil. In particular, without additional N fertilization (N0), SWC.s and SWC.h were significantly lower following CT under N0 conditions in comparison to NT over the two years of experimentation (Table 4). The absence of tillage is known to preserve soil moisture [65,76] by maintaining total soil pore space while keeping the exchanges between the macro- and micro-pores in the soil [77]. It has been shown that soil water retention under NT conditions is beneficial to the crop, notably during the grain filling period after anthesis [78]. During this period, N remobilization largely depends on soil water availability [79–81]. In line with these observations, it has been reported that in wheat both N uptake and N remobilization and thus NUE were reduced when there was a shortage of water [82,83]. It is likely that in the NT system, soil water retention was one of the components that favored post-anthesis N uptake and thus NUE.

PCA analysis allowed a refinement of the correlations observed between NUE, and the various NUE-related traits such as NUtE, NRE and NHI and their relationship with the tillage system according to the level of N fertilization (Fig 4). The first axis clearly separated CT plots from NT plots. The second axis mainly separated N0 from N1 plots. Remarkably, NUE and NUtE were the two traits that contributed the most to the increase in NUE under NT conditions. Such an analysis thus confirmed that the no-till system had a positive impact both on NUE and NUE-related traits.

## 5. Conclusion

In the present study, a field experiment was conducted over a 4-year period to ensure that the impact of the conversion to a no-till system on NUE and NUE-related traits was rapidly and accurately monitored. Both NUE and NUE-related traits, which could not have been accurately measured using longer-term experiments, were used as markers in order to investigate the benefit of the no-till cultivation system. As in a number of previous studies [74,84–86], measurements of these traits were performed using short-term experiments in order to detect the effect of no-till at any time during the entire field experiment. Over two years of experimentation, the results showed that the use of a continuous no-till system with a cover crop is a promising way to increase the NUE of maize, and consequently to reduce both the use and the loss of N fertilizers without any yield penalty.

## Supporting Information

S1 Fig Chronological representation of crop rotation over the 4-year experiment. (NT) no-till, (CT) conventional tillage, (N0) no fertilization, (N1) N fertilization, (Ø) no cover crops.
(TIF)
