# Supplementary figures and images for: Conversion to No-Till Improves Maize Nitrogen Use Efficiency in a Continuous Cover Cropping System
Source: PLoS One. 2016 Oct 6;11(10):e0164234. doi: 10.1371/journal.pone.0164234 (PMC5053546; doi:10.1371/journal.pone.0164234)

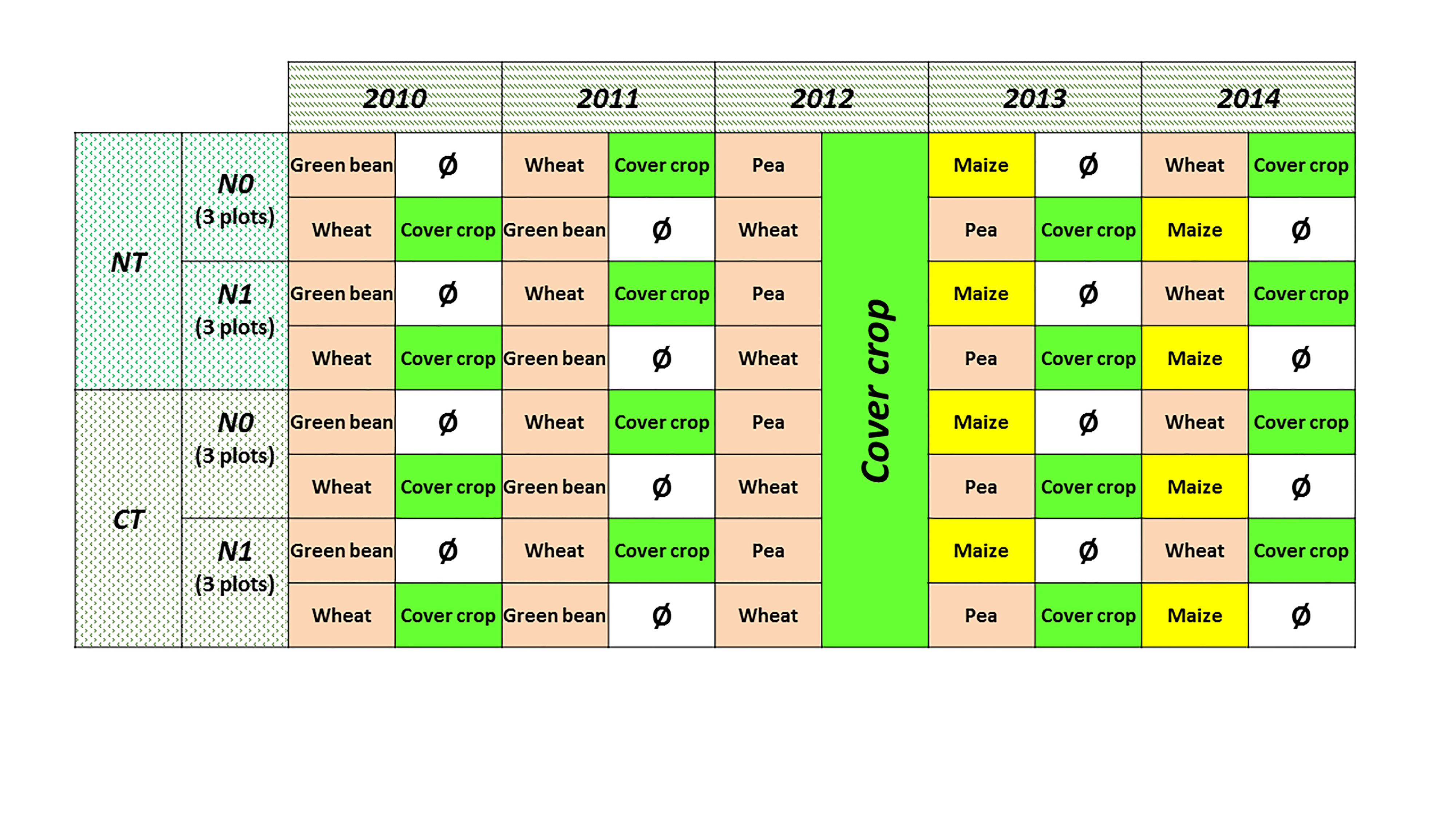

Supplement: S1 Fig — (NT) no-till, (CT) conventional tillage, (N0) no fertilization, (N1) N fertilization, (Ø) no cover crops. (TIF) [file pone.0164234.s001.tif]
